# Incomplete, atypical kawasaki disease or evolving systemic juvenile idiopathic arthritis: a case report

**DOI:** 10.4076/1757-1626-2-6962

**Published:** 2009-08-06

**Authors:** Shakeel Shaikh, Sidra Ishaque, Taimur Saleem

**Affiliations:** 1 Department of Pediatrics and Child Health, The Aga Khan University(Stadium Road), Karachi, 74800Pakistan; 2 Medical College, The Aga Khan University(Stadium Road), Karachi, 74800Pakistan

## Abstract

Kawasaki disease is an acute febrile condition seen in children. However, it is also well recognized that some patients do not fulfill the classic diagnostic criteria for the diagnosis of kawasaki disease. The incomplete form of kawasaki disease is termed as ‘Incomplete KD’ or ‘Atypical KD’. We present a case of a 6 year old child with a history of prolonged fever, periorbital, oral and lip changes, changes in the extremities and an erythamatous, maculopapular rash. Based on the physical exam and her echocardiogram that showed right coronary artery dilatation, Intravenous immune globulin was administered in this patient. This patient was refractory to two doses of intravenous immune globulin and therefore was started on methylprednisolone, to which she responded dramatically. The diagnostic dilemma primarily arose when this child presented with joint pain a day after her discharge from the hospital and a positive laboratory workup. So, was this a case of incomplete Kawasaki refractory to intravenous immuno globulin therapy or systemic juvenile idiopathic arthritis? We suggest that physicians should be cognizant of the fact that they must individualize every patient’s management to the best of their knowledge and judgment, rather than merely going by the guidelines.

## Case presentation

A 6 years and 4 months old girl from Karachi, Pakistan presented with a 15 days history of fever, sore throat, dry and cracked lips, rash and peri-orbital swelling. The fever was high grade, intermittent and associated with chills and rigors. The rash was erythematous and patchy in distribution with involvement of the face and limbs. There was swelling on the body which was initially peri-orbital and then became more generalized to involve the extremities. She also had a history of reduced oral intake since the past ten days. Associated complaints included arthralgia without ostensible arthritis, diarrhea and several episodes of vomiting. Before coming to our hospital, she had been treated with amoxicillin, lincomycin, cefixime and clarithromycin for a week by a local physician without any improvement in signs or symptoms. She had received all her vaccinations as per EPI (extended program for immunization) schedule of Pakistan.

**Figure 1.**
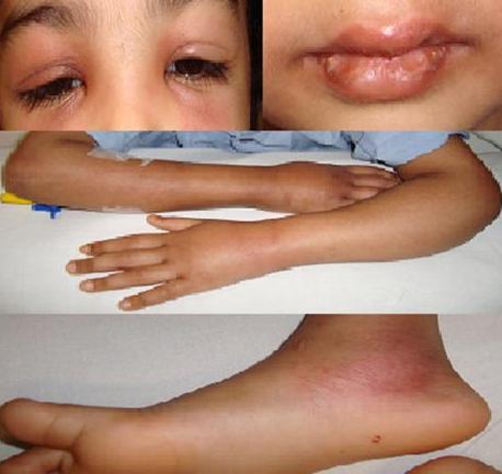
6 years old girl with peri-orbital erythema and swelling, cracked lips, swelling and rash of extremities.

On examination, she had an irritable and toxic look with bilateral peri-orbital swelling and cracked lips. A strawberry tongue was seen on examination of the buccal cavity. An erythematous, maculopapular rash on the face and limbs was observed. Edema and induration of the limbs was also appreciated. No evidence of cervical lymphadenopathy or conjunctival injection was noticed (Figure 1). She was tachcardiac with a pulse of 160 beats per minute, febrile with a temperature of 39°C. Her blood pressure was 105/66 mmHg.

With this history and presentation, the initial impression was of cellulitis, an acute hypersensitivity reaction or an incomplete kawasaki disease. She was admitted for observation and administered intravenous fluids and antibiotics (ceftriaxone and cloxacillin). Her baseline laboratory work up was sent. Abnormal laboratory findings included a low hemoglobin (9.3), a low hematocrit (28%), raised white cell count (38.4 × 10^9^/L), with a predominance of neutrophils (83.4%), and thrombocytosis (platelet count of 925). C-reactive protein and Erythrocyte Sedimentation Rate were raised (24.4 mg/dl and 100 mm/hr respectively). Albumin was lower than the normal range of 1.6 mg/dl. The rest of the work-up including electrolyte and renal function workup was within the normal range.

In addition, her blood cultures showed no growth, her urine detailed report was normal and her liver function tests showed no abnormalities.

**Figure 2.**
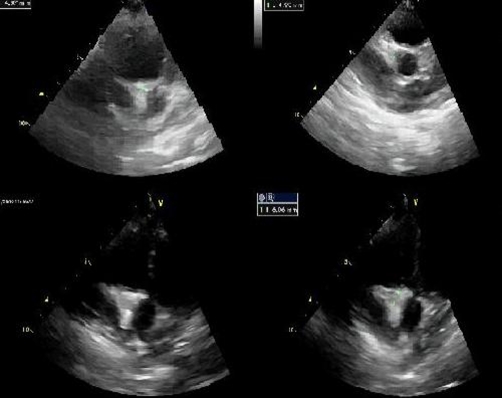
Echocardiographic images showing right coronary artery dilatation.

An echocardiogram was performed which showed a small patent foramen ovale (PFO), a normal left coronary artery and right coronary artery dilatation with thrombus formation; with ostium measurements of 5.7 - 6.2 mm and measurements in the remaining artery between 3.2 - 4 mm (Figure 2).

Based on the constellation of these findings, a diagnosis of incomplete kawasaki disease was made. She was given intravenous immunoglobulins (IVIG - 2 gm/kg), aspirin (100 mg/kg/day) and acetaminophen as needed. However, the response to this management was sub-optimal. She continued to have persistent fever spikes; even after > 36 hours of the completion of the first dose of IVIG and her inflammatory markers remained elevated with a C-reactive protein of 25 mg/dl and platelet count of 1365 × 10^9^/L. She was then given a second dose of IVIG. Her autoimmune profile was also sent at this stage which was negative (ANA, ASMA, AMA). She was given pulse therapy with intravenous methylprednisolone for three days after her failure to respond to the second dose of IVIG. In addition, she received clopidogerel, aspirin, ceftriaxone and vancomycin. She showed significant clinical improvement after institution of steroid therapy with resolution of fever spikes and normalization of inflammatory markers (ESR = 55, CRP = 3.7 and platelet = 660 × 10^9^/L). Repeat echocardiography showed no change as compared to previous study.

During the hospital stay, she developed the complaint of generalized abdominal pain. An ultrasound of the abdomen was performed which showed mild hepatomegaly with some echogenic areas in the renal parenchyma in both kidneys. This most likely represented protein casts. She was discharged from the hospital on the 13^th^ day of admission on proton pump inhibitors, clopidogerel and aspirin.

However, one day after her discharge from the hospital, she returned to the emergency room with complaints of high grade fever and backache for one day. On examination, she had arthritis of the right knee joint and right hip joint. No abnormalities of the palms, soles and oral mucosa were noted and no rash or desquamation was seen. She was admitted and started on intravenous fluids, antibiotics and NSAIDs for relief of arthritis. Anti-nuclear antibody and Rheumatoid Factor were also sent which came back to be borderline high. An aortogram was also done to better visualize the coronary arteries; dilatation of the right coronary artery was not seen on this study. A diagnosis of evolving Systemic Juvenile Idiopathic Arthritis was made because of the short time duration (< 6 weeks). She was started on steroids, proton pump inhibitor, naproxen and clopidogerel and discharged after an uneventful stay of 6 days. Her follow-up in the clinic after a week showed improvement with a CRP of 0.3 mg/dl, platelets of 668 × 10^9^/L, ESR = 5 mm/hr and Hemoglobin of 13.6 g/dl).

She has been improving clinically over the last few months. All her recent follow ups have been unremarkable in terms of any fever, joint pain, swelling or limitation of activity. The child is healthy, active and playful with no active complaints. Her systemic exams have also been unremarkable so far. Her last EDSR and CRP were within the normal range. She has been off steroids since the last month. She was started on 4 tablets of prednisone, which has been tapered down to zero over the last five months. Her current therapy comprises of Tab Naproxen Sodium (once daily).

## Discussion

Kawasaki disease (KD) is an acute febrile condition seen in children. Even though it was first reported in Japan about 30 years ago, the original diagnostic criteria defined by Dr Tomisaku Kawasaki in 1967 are still authentic and widely used today. However, it is also well recognized that some patients do not fulfill the classic diagnostic criteria for the diagnosis of KD. The incomplete form of KD is termed as ‘Incomplete KD’ or ‘Atypical KD’. Because incomplete KD is not a mild form of KD, children remain at similar risk for cardiovascular sequelae as that of complete KD [1]. Since the disease has a similar risk of coronary artery abnormalities (CAA) as complete KD [2-5], it is necessary to make an accurate diagnosis in order to prevent the development of coronary artery abnormalities CAA [1-4]. “Incomplete” KD is the preferred term, as these patients do not appear to differ from those with classic KD in any way except that they lack a sufficient number of criteria to fulfill the epidemiologic case definition [5]. The American Heart Association guidelines have been used for the diagnosis of incomplete KD, which is based on echocardiographic findings and laboratory findings [6].

Cervical lymphadenopathy is the cardinal manifestation most often absent in children with incomplete KD as was observed in our patient [1], this is followed by rash, peripheral extremity and mucous membrane changes [7]. Because of the prolonged unexplained history of fever, the typical age group of the patient and the rash, incomplete KD was considered in our patient.

The guidelines for the evaluation and treatment of patients in whom incomplete KD is suspected were established by the American Heart Association in 2004. [6] Our patient had a 15 day history of fever and three of the principal clinical criteria. Clinical signs and symptoms were consistent with KD. According to the algorithm of the guidelines, assessment using laboratory tests was required [1]. The tests showed that the patient had a C-reactive protein level of ≥3.0 mg/dL and fulfilled ≥3 supplemental laboratory criteria (albumin ≤3.0 g/dL, anaemia for age, platelets after 7 days ≥45 × 104/μL, WBC counts ≥15 000/μL and urine ≥10 WBCs/ high-power field). Therefore, we considered this patient to be suffering from incomplete KD.

She was administered the first dose of IVIG, keeping in mind her clinical presentation and laboratory parameters. Data supports the use of IVIG while there is ongoing inflammation (usually taken as ongoing fever or raised acute inflammatory markers. One high quality systemic review of sixteen RCTs showed that IVIG is of benefit in treating kawasaki disease. [19]. In children with fever and classic clinical and laboratory findings of KD, treatment with IVIG resulted in better coronary outcomes and decreased the total length of time of clinical symptoms [20].

Immunosuppressive therapy was administered in this case to control the symptoms after two doses of refractory IVIG treatment. There could be two considerations in the light of the stated scenario. It could have been a case of incomplete KD that was refractory to IVIG therapy, responding later to methyl prednisolone. Failure to respond usually is defined as persistent or recrudenscent fever more than 36 hours after completion of the initial IVIG infusion [8]. The infusion of intravenous immunoglobulin (IVIG) within 10 days of the onset of KD is known to reduce both the duration of fever and the incidence of coronary artery disease, and thus together with aspirin are the standard treatment [9,10]. However, approximately 15-20% of the children with KD have a persistent or recurrent fever and a progression of coronary dilatation despite IVIG treatment [8,11,12]. Studies support the use of corticosteroids in most patients with IVIG-refractory KD [13,14]. Wright et al first described the outcome in patients with IVIG-resistant KD who were treated with pulsed doses of corticosteroid. All the patients were treated successfully with pulse methylprednisolone with no adverse effects noted. A recent review article suggests that pulse methylprednisolone therapy should be considered if there is no response to two standard doses of IVIG treatment [15].

The other possibility is that this was a case of an evolving systemic juvenile idiopathic arthritis (SJIA), given the less than 6 week presentation, with fever, arthritis, rash and a positive lab workup. The main dilemma is that neither KD nor SJIA have absolutely specific diagnostic laboratory tests. Both diseases show similar laboratory findings with elevated WBC counts, elevated C-reactive protein, leukocytosis, thrombocytosis and anaemia and these account for diagnostic confusion. Moreover, the initial presentation of SJIA appears similar to incomplete KD. Prolonged fever and erythematous rash are common findings in both incomplete KD and SJIA [16]. These findings suggest that SJIA cannot be easily distinguished from incomplete KD. In our case, the clinical and diagnostic dilemma primarily arose when the patient presented with joint pain a day after her discharge from the hospital and a positive laboratory workup [16].

The echocardiogram plays a key role in making a diagnosis of incomplete KD. However, aneurysms rarely form before the 10th day of illness [17,18]. However, instituting IVIG therapy within the first 10 days of illness is recommended [16].

Therefore, physicians are always in a dilemma over whether to first make an accurate diagnosis or begin early treatment to prevent the development of CAA. IVIG therapy is not only a costly intervention but it also exposes the patients to unnecessary risks related to receiving a blood product [18], the diagnosis of incomplete KD requires sufficient clinical evidence. Although an aortogram done later in our patient showed no abnormal findings, her clinical features, laboratory findings and echocardiographic findings were sufficiently strong evidence to warrant a working diagnosis of incomplete KD and therefore IVIG was administered.

It is important to recognize that as long as the diagnosis of KD is based upon clinical criteria, it will remain a deficient undertaking. As long as the disadvantages of under-treating appear to outweigh the disadvantages of over-treating, IVIG will be used injudiciously to treat these children. However, this approach of treating children at risk even though the diagnosis is uncertain should result in fewer children with incomplete KD going untreated and subsequently developing coronary artery aneurysms. Hopefully, with increasing knowledge, diagnostic accuracy will improve over time, thereby ensuring that proper therapy is instituted correctly in patients with KD and SJIA.

## Conclusion

We have presented here a case causing considerable diagnostic dilemma. Physicians need to recognize that systemic-onset juvenile idiopathic arthritis can be mistaken for incomplete kawasaki disease, despite the use of established guidelines.

## Abbreviations

AMA: Anti mitochondrial antibody
ANA: Anti nuclear antibody
ASMA: Anti Smith antibody
CAA: Coronary artery aneurism
CRP: C- reactive protein
ESR: Erythrocyte sedimentation rate
IVIG: Intravenous immune globulin
KD: kawasaki disease
NSAIDS: Non steroidal anti inflammatory drugs
SJIA: Systemic juvenile idiopathic arthritis
